# Environmental impact of menstrual hygiene products

**DOI:** 10.2471/BLT.24.291421

**Published:** 2024-11-04

**Authors:** Mandip Aujla, Carmen H Logie, Anita Hardon, Manjulaa Narasimhan

**Affiliations:** a Department of Sexual and Reproductive Health and Research, World Health Organization, Avenue Appia 20, 1211 Geneva, Switzerland.; b Factor-Inwentash Faculty of Social Work, University of Toronto, Toronto, Canada.; c Knowledge, Technology and Innovation, Wageningen University and Research, Wageningen, Kingdom of the Netherlands.

Manufactured single-use pads and tampons were initially met with public distrust, but over time have replaced home-made sanitary napkins as the standard methods of menstrual care in high-income settings. In this article, we explore the environmental impacts of menstrual hygiene products and alternative options.

## Menstrual equity and the market

In the modern era of commerce and mass consumption, purchasing decisions regarding menstrual products are influenced by availability, comfort, duration of wear, cost, ease of use, reliability and peer influence. However, not every menstruator has the luxury of choice regarding menstrual hygiene management. The World Bank estimates that 500 million women lack access to menstrual products and appropriate facilities for menstruation management, leading to harmful educational and socioeconomic consequences. Research and policy attention in the past two decades has been directed to tackling the global crisis of period poverty.^1^ One approach to this problem is removal of the value-added tax levied on pads and tampons, the so-called tampon tax. The Kenyan government became the first to eliminate this tax in 2004. Several countries followed suit, and 48 countries currently have laws eliminating or reducing tax on period products. In 2021, the Scottish government became the first to provide free period products, including sustainable options, such as reusable menstrual cups and cloth pads to all who need them. Although not a tax incentive, the government of New Zealand amended its policy for access to free menstrual products in schools, including environmentally friendly options for young people.

Following the United Nations (UN) Water Conference 2023, women and feminist organizations pledged to continue advocating for national menstrual health policies under the UN Menstrual Health and Dignity Commitment, pointing out that only around 40 countries are reporting on menstrual health management. The commitment’s members also add that even fewer governments are working on promoting menstrual health products that are better for the environment and climate, can be reused and are free of toxic chemicals.^2^ While policy attention to increase access, affordability and availability of period products has increased, policy-makers have given less attention to the environmental impact of single-use period products.

## Environmental impacts

Single-use pads and tampons remain the most popular method of menstrual care in high-income countries today. The global market for manufactured menstrual hygiene products was worth 22 billion United States dollars (US$) in 2022.^3^ In the European Union alone, 49 billion single-use products are consumed annually.^4^ A person can expect to use approximately 10 000 menstrual products in their lifetime.^5^ Since their initial commercial introduction, tampons and pads have constantly evolved as new synthetic materials were invented, and are now made of over 90% plastic.

Estimation of the environmental impacts of single-use menstrual products include the use of raw materials, energy and water during manufacturing, product ingredients, packaging and disposal of waste. In the United Kingdom of Great Britain and Northern Ireland alone, over 200 000 metric tons of waste are generated from period products annually.^6^

Despite the presence of organic matter, used menstrual products are not classified as medical waste, and recycling them is economically and technically challenging. When waste from single-use menstrual products is collected separately, it is sent for incineration or landfill, depending on local waste management policies. In Europe and the United States of America, over 80% of menstrual products end up in landfills, where disposable pads may take 500–800 years to break down.^7^ Pads and tampons that are not thrown out as solid waste typically enter water systems. For instance, in the United Kingdom alone, 2.5 million tampons and 1.4 million pads are flushed daily – their super-absorptive materials absorb water, resulting in sewage backflow. Where menstruation is stigmatized and formal sanitation disposal systems lacking, pads and tampons may be disposed of in informal waste dumps and pit latrines, buried or thrown into nearby rivers.

The lack of consensus on how to classify, discard and dispose of single-use menstrual products continues to hamper efforts to tackle their environmental impacts. In May 2023, the World Health Assembly adopted a resolution on the impact of chemicals, waste and pollution on health, to end plastic pollution, expressing concern “that the production, consumption and disposal of plastic products, including microplastics and related chemicals, which can be released to the environment, may potentially impact human, plant and animal health as well as the environment, directly or indirectly.”^8^ This issue is at the heart of the Intergovernmental Negotiating Committee on Plastic Pollution’s ongoing dialogues for a Globally Binding Plastic Treaty. In preparation for the fifth session in November 2024, the International Hub Against Plastic Pollution called attention to the polluting impact of single-use plastics, noting that annual production and use of 12 billion disposable menstrual management products globally contributes an estimated 245 000 tonnes of carbon dioxide annually.^9^ Furthermore, tampons and menstrual pads contain potentially toxic chemicals such as cadmium, lead and mercury, and bleaching chemicals that can be absorbed in the users’ bodies, increasing risks of kidney, cardiovascular and inflammatory diseases.^5^

With growing awareness of the environmental burden of single-use products, menstrual activists have been promoting a return to sustainable, ecologically sound alternatives. The UN Menstrual Health and Dignity Commitment members support local production of reusable menstrual health and hygiene products, creating jobs and access, particularly in rural areas. Reusable pads, menstrual underwear, menstrual discs, menstrual cups and biodegradable single-use pads and tampons are examples of alternatives to single-use pads and tampons that can reduce the amount of plastic in the environment. These items have other benefits as well; for instance, menstrual cup use revealed multiple benefits for the user, including lower stress regarding stains and leaks, increased mobility, increased school attendance and monthly cost savings.^10^ Furthermore, in response to consumer demand, companies are increasingly offering biodegradable menstrual products that are free from pesticides, fragrances and harmful chemicals. But these alternatives are not a panacea for all people or the planet. Biodegradable and reusable products may have higher upfront production costs than single-use disposable products. Organic single-use alternative menstrual products can be resource-intensive to produce; for example, cotton crops require more water to grow relative to other crops, and non-organic cotton is often saturated in pesticides and insecticides, which is another environmental concern. Lastly, reusable products require the user to have access to clean water, soap and a private space to dry for re-use.

## Stigma

Beyond access to sustainable menstrual products, menstruation management provides a gateway to advocate for broader sexual and reproductive health and rights and bodily autonomy issues. For many, menstruation is stigmatized. Stigma processes of othering (that is, where certain individuals or communities are described, perceived and treated as different than oneself or what society classifies as normal) and devaluation have been applied to menstrual blood that may be viewed as unclean and in turn be perceived as a blemish on one’s character.^11^ In many contexts, menstrual blood is socially constructed – under unfounded beliefs – as dirty, impure and distinct from other body fluids.^12^ This menstrual stigma can be internalized in some contexts, resulting in discomfort and disgust handling bloodied products, and anticipated stigma, producing concerns regarding the visibility of washing and publicly drying reusable period products. While the environmental effects of single-use products must be remedied through alternative menstruation management solutions, people who use single-use menstrual products should not be further stigmatized for doing so, as many people have limited affordable, available, accessible and culturally and contextually relevant options.

## Conclusion

The phenomenon of single-use menstrual products, which emerged in the 20th century and were marketed as convenient and hygienic, represents a powerful example of using technological solutions to manage a natural occurrence. While single-use menstrual products provide agency to women and girls, they also contribute to harmful plastic pollution. Governments should make access to acceptable, affordable and quality menstrual health products a priority. Policy-makers should make efforts to measure, assess and reduce the planetary impacts of single-use menstrual products. Gathered evidence will support consumers and decision-makers to opt for the least environmentally harmful menstrual product option within their socioeconomic contexts.
